# The experience of lived space in persons with dementia: a systematic meta-synthesis

**DOI:** 10.1186/s12877-018-0728-0

**Published:** 2018-02-01

**Authors:** Linn Hege Førsund, Ellen Karine Grov, Anne-Sofie Helvik, Lene Kristine Juvet, Kirsti Skovdahl, Siren Eriksen

**Affiliations:** 1 Department of Nursing and Health Sciences, Faculty of Health and Social Sciences, University College of Southeast Norway, Postbox 7053, N- 3007 Drammen, Norway; 2 Department of Nursing and Health Promotion, Faculty of Health Sciences, Oslo Metropolitan University, Oslo, Norway; 3 Norwegian National Advisory Unit on Ageing and Health, Tønsberg, Norway; 4 Department of Public Health and General Practice, Norwegian University of Science and Technology, Trondheim, Norway; 5 The National Institute of Public Health, Oslo, Norway

**Keywords:** Dementia, Meta-synthesis, Interviews, Space, Place, Home, Long-term care, Life world perspective, Person’s experiences

## Abstract

**Background:**

Identifying how persons with dementia experience lived space is important for enabling supportive living environments and creating communities that compensate for the fading capabilities of these persons. Several single studies have explored this topic; however, few studies have attempted to explicitly review and synthesize this research literature. The aim of this systematic meta-synthesis was therefore to interpret and synthesize knowledge regarding persons with dementia’s experience of space.

**Methods:**

A systematic, computerized search of AgeLine, CINAHL Complete, Embase, Medline and PsycINFO was conducted using a search strategy that combined MeSH terms and text words for different types of *dementia* with different descriptions of *experience.* Studies with 1) a sample of persons with dementia, 2) qualitative interviews as a research method and 3) a description of experiences of lived space were included. The search resulted in 1386 articles, of which 136 were identified as eligible and were read and assessed using the CASP criteria. The analysis was inspired by qualitative content analyses.

**Results:**

This interpretative qualitative meta-synthesis included 45 articles encompassing interviews with 672 persons with dementia. The analysis showed that *living in one’s own home* and *living in long-term care* established different settings and posed diverse challenges for the experience of lived space in persons with dementia. The material revealed four main categories that described the experience of lived space: (1) *belonging*; (2) *meaningfulness*; (3) *safety and security*; and (4) *autonomy*. It showed how persons with dementia experienced a reduction in their lived space due to the progression of dementia. A comprehensive understanding of the categories led to the latent theme: “Living with dementia is like living in a space where the walls keep closing in”.

**Conclusion:**

This meta-synthesis reveals a process whereby lived space gradually becomes smaller for persons with dementia. This underscores the importance of being aware of the experiences of persons with dementia and the spatial dimensions of their life-world. To sustain person-centred care and support the preservation of continuity and identity, one must acknowledge not only the physical and social environment but also space as an existential experience for persons with dementia.

## Background

Living with dementia involves enduring the loss of several mental and physical abilities [1], which leads to difficulties in handling everyday living [2], maintaining meaningful activities [3] and taking part in social life [4, 5]. Globally, at least 46.8 million people are living with dementia, and the number is rapidly rising [6]. As dementia is the leading cause of dependency and frailty among older people, delivering sufficient care services for persons with dementia constitutes one of the greatest challenges within health care systems [7].

Shifts in the perspectives of dementia care are urgently needed; this includes a shift in focus from symptoms and the disability and towards the capacities and potential of persons with dementia and their families [8, 9]. The World Health Organization’s “age-friendly” policy movement [10] and dementia awareness campaign [7] remind the general public of the importance of empowering persons with dementia to remain autonomous and active citizens of society. Understanding how persons with dementia experience the spatial dimensions of their day-to-day experiences of living with dementia is therefore important. This is necessary to both enable supportive living environments and create communities that compensate for the fading abilities of persons with dementia and allow them to maintain a meaningful life [9, 11]. Dutch philosopher Max Van Manen [12] described lived space as one of four existential attributes that he believed could guide reflections regarding the constitution of people’s lifeworld. These four attributes are (1) lived body, (2) lived others, (3) lived time, and (4) lived space. He conceptualized lived space as felt space and as a category for inquiring into the ways in which people experience the spatial dimensions of their daily experiences [12]. Thus, lived space, as conceptualized in this review, is more than the spatial characteristics of space and its geographies; it is also related to the feeling of being home or the conceptualization of “being in place” [13–15]. It refers to the meaning of space in relation to the experiences of living with dementia.

Because of the magnitude of its consequences, dementia is a syndrome that often requires individual care approaches and facilitated living environments [16]. Growing evidence suggests that housing- and environmental-design, in combination with psychosocial interventions, may have a positive impact on the functional level and quality of life of people with dementia [17–19]. These types of facilitating approaches are often described in the research literature and in policy documents through the concept of dementia-friendly or dementia-capable environments [20–23]. Person-centred care is commonly referred to as an important care approach [1, 24] that may promote this type of sustaining environment. Individualized care, recognition of the perspectives of the person with dementia, and the creation of social environments that support the well-being of the person are viewed as important cornerstones of person-centred care [25]. Although person-centred care has valuably influenced the development of dementia care, scholars have also called for more emphasis on how the physical and social environment can be adapted to support the preservation of continuity and identity for persons with dementia [26, 27].

Among the central principals for the design of dementia-friendly environments are safety and security, simplicity, good structure, and familiarity [11, 18, 28, 29]. Simple, structured and familiar environments may support wayfinding [21, 29–31]. The concept of familiarity refers to predictability and continuity; it is also important for creating a sense of being at home for persons with dementia in addition to supporting the maintenance of their social relationships, identity, autonomy and privacy [11, 32–35]. These are other important elements of the experience of lived space.

Although the majority of the existing guidelines describing design interventions for persons with dementia are founded on research conducted in institutional settings, similar design requirements are regarded as relevant for private homes and for accommodating the continued use and navigation of the outdoor environments [11, 21]. However, the guidelines largely reflect the spatial characteristics of the environment rather than reflecting the spatial dimensions and the feelings among persons with dementia regarding the use of the environment.

Identifying how persons with dementia might experience lived space is important not only to enable the physical environments and to compensate for their incapacities but also to facilitate their continuous engagement and activity in society. Several single studies have explored the perspectives of persons with dementia regarding how they experience lived spaces in different contexts, for example, the process of moving into residential care [36–38], living with dementia in long-term care [39–43], receiving community-based home care services [44] and living alone while having dementia [45–49]. Others have described the perspectives of lived space by investigating the experience of meaningful activity and the important aspects of life [43, 50–52], the use of everyday technology [53], the experience of the outdoor environment [54] and the accessibility to public space [55]. Literature on the experiences of lived space for persons with dementia is comprehensive and includes several different perspectives, but to our knowledge, few studies have attempted to explicitly review and synthesize this body of literature. Considering how important lived space is for identity and meaningful activities, there is a need for a systematic review that synthesizes the knowledge on this topic. Therefore, the aim of this systematic meta-synthesis was to interpret and synthesize the experience of lived space for persons with dementia.

## Methods

### Design

The research group conducted an interpretative qualitative meta-synthesis, inspired by the approaches and methods described by Paterson and colleagues and Zimmer [56, 57].

### Search method

**Fig. 1:**
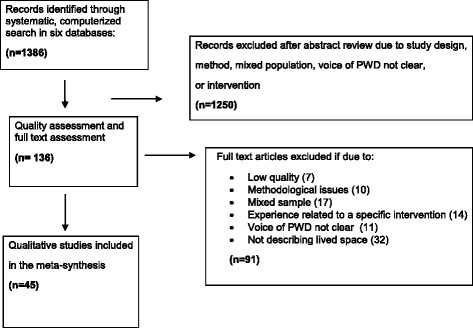
Flow chart of the literature search

A systematic and computerized search of AgeLine, CINAHL Complete, Embase, Medline and PsycINFO were conducted . MeSH terms and text words for different types of *dementia* were combined with different descriptions of *experience.* The combinations of the search terms are shown in Table 1. Studies were limited to qualitative, peer-reviewed research articles of high methodological quality written in English and published between January 2004 and March 2017. The searches resulted in 1386 articles, of which 136 were identified as eligible. Figure 1 presents a flow chart for the selection of the articles.

**Table 1:** Search terms

Population	Experience
MeSH terms:	MeSH terms:
• Dementia (CINAHL, Embase, Medline, PsycINFO)	• Life experience (CINAHL, PsycINFO)
• Dementia, presenile (CINAHL, Embase, Medline, PsycINFO)	• Experience (Embase)
• Dementia, senile (CINAHL, Embase, Medline, PsycINFO)	• Personal experience (Embase)
• Alzheimer’s disease (CINAHL, Embase, Medline, PsycINFO)	
• Dementia, multi-infarct (CINAHL, Embase, Medline, PsycINFO)	
• Lewy Body Disease (CINAHL, Embase, Medline, PsycINFO)	
• Dementia, vascular (CINAHL, Medline, PsycINFO)	
• Dementia, frontotemporal (Embase)	
Text words:	Text words:
• Dement*	• Personal experience*
• Presenile dement*	• Experience*
• Senile dement*	• Lived experience*
• Alzheimer*	• Life experience*
• Multi-infarct dement*	• Patient experience*
• Lewy Body dement*	• Subjective experience*
• Vascular dement*	• First-person
• Frontotemporal dement*	• **
All words combined with OR	All words combined with OR
	**Ageline had no exckusion parametes for clinical queries, and we had to search for study design: nursing methodologies OR case study OR constant comparison OR content analysis OR descriptive study OR discourse analysis OR ethnography OR exploratory OR feminist OR grounded theory OR hermeneutic OR interview OR narrative OR naturalistic OR participant observation OR phenomenology OR qualitative research OR qualitative methods OR qualitative study.

To ensure that there were always at least two authors selecting the articles in terms of screening, eligibility and inclusion, three pairs of authors (EKG and SE/LKJ and SE/LHF and SE) autonomously reviewed the titles, the abstracts, and, in some cases, the full articles of all of the identified studies. This was in accordance with the PRISMA standard of systematic reviews [58]. The authors resolved disagreements by discussion and, if needed, by consulting one of the other pairs of authors. The studies were included if they comprised the following components: (1) a sample of persons with dementia only; (2) a qualitative interview as a research method; (3) the explicit voices of persons with dementia; and (4) a description of experiences of the lived space. The exclusion criteria were as follows: (1) they used a mixed sample, or the dementia diagnosis of the sample was uncertain (i.e., probable or possible dementia), or (2) they studied a certain intervention.

### Assessment of the quality of the studies

One hundred thirty-six full text articles were read and reviewed according to the Critical Appraisal Skills Programme (CASP) criteria for qualitative studies [59]. Pairs of authors (LKJ/EKG, KS/LHF, SE/ASH or EKG/SE) assessed the quality of all of the studies. The authors resolved disagreements by discussion and, if necessary, by consulting one of the other pairs of authors. The CASP appraisal tool includes the following nine criteria: (1) a precise statement of aims; (2) an applicable choice of method; (3) a suitable research design; (4) congruence between the recruitment strategy, aims and research; (5) the methods for data collection addressed the research issue; (6) the relationship between the researcher and the participant was considered; (7) ethical issues were reflected; (8) the process of data analysis was sufficiently rigorous; and (9) a clear statement of the findings. An equal weight (i.e., 1 point) was given each criterion, and maximum score was nine for each quality assessment of the studies. A score of 9 points indicated high methodological quality, whereas 7-8 points indicated moderate quality (see Table 2). This review only included studies with moderate and high quality. Seven studies had scores <7 and were therefore excluded due to low quality. This meta-synthesis is based on 45 articles; the studies included are presented in Table 3.

**Table 2:** Quality assessment of studies included

Study	Criteria^a^									Total /9	Quality
	1	2	3	4	5	6	7	8	9		
Aminzadeh [36], 2009	+	+	+	+	+	+	+	+	+	9	High
Aminzadeh [37], 2010	+	+	+	+	+	-	-	+	+	7	Moderate
Beattie [90], 2004	+	+	+	+	+	+	+	+	+	9	High
Bronner [91], 2016	+	+	+	+	+	-	-	+	+	7	Moderate
Brorsson [55], 2011	+	+	+	+	+	+	+	+	+	9	High
Clare [92], 2008	+	+	+	+	+	+	+	+	+	9	High
De Witt [49], 2009	+	+	+	+	+	+	+	+	+	9	High
De Witt [48], 2010	+	+	+	+	+	+	+	+	+	9	High
Digby [93], 2012	+	+	+	+	+	+	+	-	+	8	Moderate
Digby [94], 2014	+	+	+	+	+	+	+	+	+	9	High
Duggan [54], 2008	+	+	+	+	+	+	+	-	+	8	Moderate
Fleming [34], 2015	+	+	+	+	+	+	+	+	+	9	High
Frazer [47], 2012	+	+	+	+	+	+	+	+	+	9	High
Gill [44], 2011	+	+	+	+	+	+	+	+	+	9	High
Gilmour [95], 2005	+	+	+	+	+	+	+	+	+	9	High
Goodman [96], 2013	+	+	+	+	+	-	+	+	+	8	Moderate
Harmer [26], 2008	+	+	+	+	+	+	+	+	+	9	High
Hedman [42], 2013	+	+	+	+	+	+	+	+	+	9	High
Heggestad [66], 2013	+	+	+	+	+	+	+	+	+	9	High
Hulko [97], 2009	+	+	+	+	+	+	+	+	+	9	High
Johannessen [98], 2011	+	+	+	+	+	-	+	+	+	8	Moderate
Keller [63], 2010	+	+	+	+	+	+	-	+	+	8	Moderate
Lawrence [99], 2011	+	+	+	+	+	+	+	+	+	9	High
Liou [65], 2013	+	+ ++	+	+	+	+	+	+	+	9	High
Mazaheri [100], 2014	+	+	+	+	+	+	+	+	+	9	High
Mjorud [41], 2017	+	+	+	+	+	+	+	+	+	9	High
Mok [101], 2007	+	+	-	+	+	+	-	+	+	7	Moderate
Molyneaux [102], 2012	+	+	+	+	+	+	+	+	+	9	High
Moyle [43], 2011	+	+	+	+	-	-	+	+	+	7	Moderate
Mushi [103], 2014	+	+	-	+	+	+	+	+	+	8	Moderate
Nowell [67], 2013	+	+	+	+	+	+	+	+	+	9	High
Nygård [45], 2008	+	+	+	+	+	-	-	+	+	7	Moderate
Öman [104], 2005	+	+	+	+	+	-	-	+	+	7	Moderate
Pesonen [105], 2013	+	+	+	+	+	+	+	+	+	9	High
Phinney [50], 2006	+	+	+	+	+	-	+	+	+	8	Moderate
Phinney [51], 2011	+	+	+	+	+	+	+	-	+	9	Moderate
Pipon-Young [106], 2012	+	+	+	+	+	+	+	+	+	9	High
Rostad [107], 2013	+	+	+	+	+	-	+	+	+	8	Moderate
Samsi [108], 2013	+	+	+	+	+	-	+	+	+	8	Moderate
Svanström [46], 2015	+	+	+	+	+	-	+	+	+	8	Moderate
Tak [39], 2014	+	+	+	+	+	-	+	+	+	8	Moderate
Thein [38], 2011	+	+	+	+	+	-	+	-	+	7	Moderate
Van Zadelhoff [40], 2011	+	+	+	+	+	-	+	+	+	8	Moderate
Vikström [64], 2008	+	+	+	+	+	-	-	+	+	7	Moderate
Wolverson [52], 2010	+	+	+	+	+	+	-	+	+	8	Moderate

1. Clear research statement, 2.Qualitative methodology, 3.Research question appropriate, 4.Recruitment strategy, 5.Data collection, 6.Relationship researcher – participants described adequately, 7.Ethical considerations, 8.Data analysis, 9.Clear statements of findings

9 = high quality, 7-8 = moderate quality, 6 or less = low quality

^a^CASP criteria

**Table 3:** Presentation of studies included

Authors & year	Aim	Participants	Method
Aminzadeh F, Dalziel WB, Molnar FJ & Garcia LJ [36], 2009	To explore the subjective meaning of relocation for persons with dementia moving into residential care	N=16 persons diagnosed with dementia living at home and planning to move to residential care within 2 months. Canada	Individual in-depth interviews. Field notes as supplementary data. Analyses were guided by the work of Corbin & Strauss.
Aminzadeh F, Dalziel WB, Molnar FJ & Garcia L [37], 2010	To examine the significance of home at the time of relocation to residential care from the perspective of persons with dementia	N=16 persons diagnosed with dementia living at home and planning to move to residential care within 2 months. Canada	Individual in-depth interviews. Field notes as supplementary data. Analyses were guided by the work of Corbin & Strauss.
Beattie A, Gavin D-W, Gilliard J & Means R [90], 2004	To demonstrate how interviews can be conducted with younger people with dementia.	N=14 participants who had received a diagnosis of dementia and were using services. UK	Semi-structured, individual in-depth interviews Data were transcribed and subjected to comparative textual analysis guided by the principles of Strauss & Corbin
Bronner K, Perneczky R, McCabe R, Kurz A & Harmann J [91], 2016	To identify medical and social topics which become relevant in the period following diagnosis of AD, for which a decision may eventually need to be made and which has implications for the life and wellbeing of the persons with AD	N= 5 persons with AD, relatives (6) and professionals (13). Germany	Semi-structured face-to-face interviews. Data were analysed using content analysis in accordance with Mayring.
Brorsson A, Øhman A, Lundberg S. & Nygård L [55] ,2011	To illuminate experiences of accessibility in public space in people with AD, with particular focus on placed, situations and activities that they found to be important for daily life	N= 7 persons diagnosed with early AD, living in ordinary housing Sweden	Repeated in-depth interviews. All, except for one informant, were interviewed twice. Data were analysed using open coding in accord with Corbin and Strauss.
Clare L, Rowland J, Bruce E, Surr C & Downs M [92], 2008	To explore the subjective experience of living with dementia in residential care and to understand the psychological impact of being in this situation	N= 81 persons diagnosed with dementia living in residential care homes. UK	An existing dataset consisting of individual unstructured conversations with people with dementia from a study of well-being in residential care were used. The number of conversations recorded with each participant ranged from 1-8. The total dataset consisted of 304 transcripts. Interpretative phenomenological analysis as guiding design.
De Witt L, Ploeg J & Black M [48], 2010	To understand the meaning of living alone from the perspective of older people with Alzheimer disease or a related dementia.	N = 8 women diagnosed with mild to moderate AD or related dementia living alone in the community. Canada	Repeated face-to-face, open-ended interviews. All, except for two informants, were interviewed twice. Data were analysed using three techniques data analysis in accord with van Manen.
De Witt L, Ploeg J & Black M [49], 2009	To understand the meaning of living alone for older people with dementia	N = 8 women diagnosed with mild to moderate AD or related dementia living alone in the community Canada	Repeated face-to-face, open-ended interviews. All, except for two informants, were interviewed twice. Data were analysed using three techniques data analysis in accord with van Manen.
Digby, R., Moss, C. & Bloomer, M.J. [93], 2012	To understand how older patients with mild to moderate dementia experienced the transfer from acute to subacute care and settling-in period.	N= 8 persons with dementia staying in a sub-acute facility Australia	In-depth semi-structured interviews using specific communication techniques. Data were analysed using content analysis in accord with Hsieh and Shannon.
Digby R & Bloomer MJ [94], 2014	To elicit the perspectives of current inpatients with dementia, and their family carers, about the environment/design features that they believe are necessary for people with dementia, and their family carers.	N= 7 persons with dementia staying in a sub-acute facility and carers (4) Australia	In-depth semi-structured interviews
Duggan S, Blackman T, Martyr A & Van Schaik P [54], 2008	To explore the use of outdoor environment and how dementia impacts on it.	N= 22 persons diagnosed with early to moderate AD or vascular dementia living in their own home, and carers (11 spouses/partners, 2 daughters, 1 carer/housekeeper) UK	Semi-structured individual interviews. Data were analysed using NVivo and further in line with grounded theory.
Fleming R, Kelly F & Stillfried G [34], 2015	To identify the environmental features that are desirable in buildings used to provide care for people with dementia nearing the end of their lives	N= 2 persons with young onset dementia, family carers (10) and health care personnel (5). Australia	Mixed method. Three focus group interviews. In addition, a survey with experts in environmental design of care facilities for older people (21). Interview data were analysed using management software NVivo 8.
Frazer SM, Oyebode JR & Cleary A [47], 2012	To explore how women who live alone with dementia see themselves and how they cope in their everyday lives	N=8 persons diagnosed with dementia (AD=5) living in their own home. UK	Individual, semi-structured interviews were performed. Data were analysed using interpretative phenomenological approach.
Gill L, White L & Cameron ID [44], 2011	To understand how people with dementia receiving community care services in their own homes, perceive interaction in the context of their service experience	N=22 persons diagnosed with dementia receiving community care services in their own home. Australia	Individual semi-structured interviews were performed. Data were analysed using thematic- and constant comparison analyses.
Gilmour JA & Huntington AD [95],2005	To explore the experiences of living with memory loss	N= 9 persons diagnosed with dementia living at home. New Zealand	Individual, semi-structured interviews using open questions were used. To assist participants, questions were provided on beforehand and many participants wrote reminder notes prior to the interview. Thematic analyses were undertaken.
Goodman C, Amador S, Elmore N, Machen I & Mathie E [96], 2013	To explore how people with dementia discuss their priorities and preferences for end-of-life care, and how this might inform subsequent discussions with family and practitioners	N= 18 persons diagnosed with dementia living in residential care homes. UK	Individual, semi-structured interviews in the form of a ‘guided-conversation’ were conducted as a part of a longitudinal mixed method study. Thematic analyses were undertaken.
Harmer BJ & Orrell M [26], 2008	To explore the experience of living with dementia with focus on what makes activities meaningful for people with dementia	N=17 persons diagnosed with dementia living in residential care homes, in addition their family caregivers (8), and staff (15). UK	Focus group design with a constructed question guide with residents, staff and relatives of the residents were performed. Mind map notes. Data were analysed using grounded theory approach with contents analysis.
Hedman R, Hansebo G, Ternestedt BM, Hellström I, Norberg A [42], 2013	To explore the use of Harré’s social constructionist theory of selfhood to describe how people with mild and moderate AD express their sense of self	N= 12 persons diagnosed with AD living in their home. Sweden	Individual, semi-structured interviews were performed. Data were analysed using phenomenological approach in accord with Harré’s theory of social constructionist.
Heggestad A, Nortvedt P, Slettebø A [66], 2013	To investigate how life in Norwegian nursing homes may affect experiences of dignity among persons with dementia	N= 5 persons diagnosed with dementia living in nursing home. Norway	Individual interviews and observations field notes were used. Data were analysed using qualitative phenomenological and interpretative hermeneutical approach in accord with Kvale & Brinkman.
Hulko W [97], 2009	To explore the experience of older people with dementia and in which way socio-culture plays a role in diverse dementia patients’ daily living	N = 8 persons diagnosed with dementia (AD=7) living in their home and their relatives (50). Canada	Series of individual in-home interviews over 1-2 month and observation sessions were used. Data were analysed in accord with grounded theory.
Johannessen A & Möller A [98], 2011	To find out how people experience living with early-onset dementia, and to assess the implications for practice and the development of further services	N= 20 young persons with a diagnosis of dementia. Norway	Individual, thematic interviews were conducted. Data were analysed in line with grounded theory according to Glaser and Strauss,
Keller HH, Martin LS, Dupuis S, Genoe R, Edward HG, Cassolato C [63], 2010	To explore the mealtimes to provide opportunity for social activity and emotional connection	N=27 participants with early to mild stage of dementia living in their home and their next of kin (28). Canada	Active interviews with both individual and dyads were performed. Data were analysed using grounded theory methodology in accord with Charmaz and team analysis.
Lawrence RM, Samsi K, Banerjee S, Morgan C, Murray J [99], 2011	The subjective reality of living with dementia from the perspective of three minority ethnic groups. Thoughts and other reactions to the diagnosis dementia	N=30 persons diagnosed with dementia living at home or in sheltered accommodations (4). UK	Individual in-depth interviews were performed. Data were analysed using grounded theory approach in accord with Glaser.
Liou CL & Jarrott SE [65], 2013	To explore the experience of people with dementia in two adult day service environments within the Taiwanese culture.	N=8 persons with dementia and staff (15) Taiwan	Semi-structured interviews.Observation. Data were analysed using a deductive-inductive approach in accord with Hung and Chaudhury.
Mazaheri M, Eriksson LE, NasraBadi AN, Sunvisson H, Heikkilä K [100], 2014	To explore the subjective experience of living with dementia among Iranian immigrants in Sweden	N=15 persons diagnosed with dementia living at home (10) or in group dwellings for people with dementia. Sweden	Individual semi-structured interviews were performed. Data were analysed using content analysis in accord with Graneheim and Lundman.
Mjorud M, Engedal K, Rosvik J, Kirkevold M [41], 2017	To investigate the personal experience of living in a nursing home over time and what makes life better or worse from the perspective of the person with dementia	N=12 persons with dementia living in nursing home care units for persons with dementia Norway	Repeated individual, unstructured interviews 3 months apart. Field observations. Data were analysed using phenomenological-hermeneutical analysis in accordance with Lindseth and Norberg.
Mok E, Lai CK, Wong FL, Wan P [101], 2007	To describe the lived experience of people with early stage dementia and their ways of coping with the illness	N=15 persons with dementia living at home China	Individual interviews were performed. Data were analysed using phenomenological approach in accord with Colaizzi.
Molyneaux VJ, Butchard S, Simpson J, Murray Cl [102], 2012	To understand ‘couple-hood’ as it is co-constructed by the couple when one partner has dementia	N=5 persons diagnosed with AD and their partner living at home. UK	The couples were interviewed simultaneously. Data were analysed using constructivist grounded theory approach in accord with Charmaz.
Moyle W, Venturo L, Griffiths S, Grimbeek P, McAllister M, Oxlade D et al. [43], 2011	To understand the factors that influence quality of life for people living with dementia in long term care, including understanding of how they perceived they were valued	N=32 persons diagnosed with dementia living in long term care. Australia	Individual, semi-structured Interviews were performed. Data were analysed in accordance with Laximancer using computer assisted concept mapping program.
Mushi D, Rongai A, Paddick SM, Dotchin C, Mtuya C, Walker R [103], 2014	To explore the socio-cultural beliefs surrounding dementia and the life experience of people with dementia and their caregivers in the Tanzania	N=41 persons diagnosed with dementia living at home and their caregivers, but only 25 persons with dementia were interviewed. Tanzania	Semi structured paired interviews (25) and individual interviews (16) with the caregiver alone were performed. Data were analysed using content analysis.
Nowell ZC, Thornton A, Simpson J [67], 2013	To understand personhood by exploring the subjective experiences of those with dementia in UK	N=7 people diagnosed with dementia living in dementia care units. UK	Individual semi-structured individual interviews were performed. Data were analysed using an interpretative phenomenological approach.
Nygård L [45], 2008	To explore how people with dementia who live alone experienced the meaning of their everyday technology, such as telephone and electronic equipment, and the use of it.	N= 8 persons diagnosed with dementia living at home. Sweden	Repeated individual interviews and observations (during 3 weeks) were performed. Two to four sessions of interviews and observations pr. person, each session lasting between 1 to 2 hours. Data were analysed using a phenomenological, hermeneutical approach.
Öhman A & Nygård L [104], 2005	To uncover and describe the meaning and motives for engagement in self-chosen daily life occupation for elderly individuals with Alzheimer’s disease dwelling in community	N=6 community-dwelling persons diagnosed with AD. Sweden	Repeated individual interviews and observations. Totally two or three times per person. A qualitative comparative analysis method was used in accord with Bogdan & Biklen.
Pesonen HM, Remes AM, Isola A [105], 2013	To explore the shared experience of dementia from the viewpoint of people with newly diagnosed dementia and their family members, and to understand how they manage their lives after the diagnosis	N= 8 persons diagnosed with dementia (AD=6) living in their home or nursing home/assisted living facility (4) and their family members (8). Finland	Conversational, low structured face-to-face interviews. Unstructured observations were conducted during the interviews; field notes were written after each interview. Descriptive analysis using grounded-theory framework and constant comparative analysis in accord with Corbin & Strauss.
Phinney A [50], 2006	To learn more about the experiences the person with dementia and their families have in regard to meaningful activity	N= 8 persons diagnosed with AD living in their home with one family member. Canada	Repeated individual in-depth, conversational interviews with persons with dementia and one family member in line with van Manen were conducted. Data were analysed using interpretative phenomenological approach in accord with Brenner.
Phinney A [51], 2011	To understand how people with dementia understand their lives as making sense and worth living.	N= 9 persons with mild to moderate AD living in own homes Canada	Repeated in-depth conversational interviews. Participant observation.
Pipon-Young FE, Lee KM, Jones F, Guss R [106], 2012	To explore the experiences of younger persons with dementia and develop an understanding of helpful support To identify areas of the service in need for change	N=8 persons diagnosed with dementia living in their home. UK	Action research across three phases; semi-structured individual interviews and field notes were used. Data were analysed using action research; interpretative approach including thematic analysis techniques in line with Charmaz and concept mapping in accord with McNiff & Whitehead.
Rostad D, Hellzen O, Enmarker I [107], 2013	To gain understanding of the lived experience of younger persons with dementia (<65 years) who lived at home and suffered with early onset, and the meaning that could be found in their experiences	N=4 persons diagnosed with dementia living in their home. Norway	Individual, narrative individual interviews in a conversational style with broad open-ended questions were used. Phenomenological hermeneutic approach to the analysis in line with Lindseth and Nordberg.
Samsi K & Manthorpe J [108], 2013	To gain understanding of how everyday decision-making occur and change among people with dementia and carers from their perspective	N= 12 persons diagnosed with dementia living in their home and their family caregivers (12). UK	Face to face interviews 3-4 times during one year (approximately every 3-4 month) using a person-centered interviewing style were performed. Both joint and separate interviews was performed, according to the preferences of those interviewed (it may vary over time). Phenomenological study in accord with Smith using thematic analysis in line with Braun and Clarke.
Svanström R & Sundler AJ [46], 2015	To elucidate the phenomenon of living alone with dementia and having a manifest care need	N= 6 persons with dementia living in own homes. Sweden	Several conversational interviews and field notes. 32 visits with six participants. Data were analysed in accord with an in-depth phenomenological analysis.
Tak SH, Kedia S, Tongumpun TM & Hong SE [39], 2015	To describe types of current activity involvement and barriers to activities reported by nursing home residents with dementia	N= 37 nursing home residents with dementia. USA	Individual short, open-ended interviews (31) and individual in-depth interviews (6) were performed. Data were analysed in accord with descriptive, content analysis within ethnographic framework.
Thein NW, D’ Souza G, Sheehan B [38], 2011	To explore the subjective experience of people with dementia of the move to a care home.	N= 18 persons with mild to moderate dementia moving in to nursing home UK	Repeated semi-structured individual interviews before and after moving to nursing home. Systematically coding with NVivo using the headings for the interview as major codes. .
Van Zadelhoff E, Verbeek H, Widdershoven G, van Rossum E, Abma T [40], 2011	To investigate experiences of residents with dementia, their family and nursing staff in group living homes for older people with dementia and their perception of the care process	N=5 persons diagnosed with dementia living in a non-profit nursing home, in addition, residents’ family members (4) and staff (5). The Netherlands	Individual in-depth interviews with open-ended questions were performed separately with each of the participants. Observations and field notes were taken. Inductive and theoretical analysis was used.
Vikström S, Josephson S, Stigsdotter-Neely A, Nygård L [64], 2008	To identify and describe how persons with dementia and their caregiving spouses perceive their own, their spouses’ and their mutual engagements in everyday activities.	N=26 persons with dementia living in their home and their caregiving spouses (26). Sweden	Individual semi-structured individual interviews with open-ended questions were performed for PWD and caring spouse. Analysed using constant comparative method in line with grounded theory by Corbin & Strauss.
Wolverson EL, Clarke C, Moniz-Cook E [52], 2010	To investigate the subjective experience of hope of people with dementia	N= 10 persons diagnosed with AD living in their home. UK	Individual semi-structured interviews with open-ended questions were performed. Data were analysed using interpretative phenomenological approach in line with Smith.

Interviews with 672 persons with dementia are included in this meta-synthesis. The participants are described in Table 4. Studies were reviewed to identify how the severity of dementia was assessed. However, overall, it appeared that the studies’ inclusion criteria gave more weight to participants’ ability to provide informed consent and verbally articulate their experiences of living with dementia than to formal assessments of cognitive impairment. Several of the studies did not provide descriptions of the stage of dementia. Therefore, we lacked complete data with which to determine participants’ severity of dementia. However, all studies provided information about participants’ housing situation. Eleven studies included participants living in nursing homes or other care facilities, thirty-two studies included participants living at home, and two studies had mixed sample. As relocation to long-term care can serve as an indicator of dementia progression and severity, information about the housing situation was described (Table 4) and applied throughout the analysis. Individual interviews with persons with dementia constituted the main data in 41 of the studies. One study was based on interviews with dyads or pairs, and three studies were based on focus group interviews.

**Table 4:** Description of participants

Authors & year	Stage of dementia	Housing situation	Age (years)	Gender / female (%)
Aminzadeh [36], 2009	Mild to moderate stage of dementia MMSE (Mini-Mental State Examination) score: Range 15-27, Mean 23.8	Living in own home ^a^Living with someone: 31.3%	Mean age: 85.3 (76-93)	68.8
Aminzadeh [37], 2010	Mild to moderate stage of dementia MMSE score: Range 15-27, Mean 23.8	Living in own home Living with someone: 31.3%	Mean age: 85.3 (76-93 )	68.8
Beattie [90], 2004	Mild, moderate an severe stage of dementia No MMSE score provided	Living in own home (13) Living in nursing homes or other care facilities (1) Living with someone: 71.4%	Mean age: 59.43 (41-66)	Not described
Bronner [91], 2016	Mild stage of dementia MMSE score: MMSE >24 (range not provided), Mean 25.5	Living in own home	Mean age: 65	80.0
Brorsson [55], 2011	MMSE score: Range 18-30, Mean 23,1	Living in own home Living with someone: 42.9%	Age range: 63-80	71.4
Clare [92], 2008	MMSE score: Range 0-20, Mean 9,76	Living in nursing homes or other care facilities	Mean age: 83.4 (59-96)	85.0
De Witt L [48], 2010	Mild to moderate stage of AD assessed by FAST (Functional Assessment Staging system) No MMSE score provided	Living in own home Living alone: 100%	Age range: 58-87	100
De Witt L [49], 2009	Mild to moderate stage of AD assessed by FAST MMSE score not provided	Living in own home Living alone: 100%	Age range: 58-87	100
Digby [93], 2012	Mild to moderate stage of dementia MMSE score: Range 15-23, Mean 20,6	Inpatients in a sub-acute geriatric rehabilitation facility	Age range: 77-92	37.5
Digby [94], 2014	Mild to moderate stage of dementia MMSE score: Range 15-21, Mean 17,7	Inpatients in a sub-acute geriatric rehabilitation facility	Age range: 67-89	57.1
Duggan [54], 2008	Mild to moderate stage of dementia MMSE score: Range 15-29, (mean score not provided)	Living in own home	Age range: 71-84	50.0
Fleming G [34], 2015	Stage of dementia: not described MMSE score not provided	Living in own home	Not described	Not described
Frazer [47], 2012	Mild to moderate stage of dementia MMSE score: Range: 14-26, Mean 20	Living in own home Living alone: 100%	Age range: 75-89	100
Gill [44], 2011	Stage of dementia: not described MMSE score not provided	Living in own home Living with someone: 77.3%	Age range: 80- 92	63.6
Gilmour [95], 2005	Stage of dementia: not described MMSE score not provided	Living in own home Living with partner: 88.9%	Age range: 56-79	44.4
Goodman [96], 2013	Stage of dementia: not described MMSE score not provided	Living in nursing homes or other care facilities Length of stay: 3-61 months	Age range: 68-92	72.2
Harmer [26], 2008	MMSE score: Range 5-25, Mean 12	Living in nursing homes or other care facilities Living with partner: 17.6%	Mean age: 85.6 (72-99)	70.5
Hedman [42], 2013	Mild to moderate stage of dementia assessed by using the Cognitive Performance Scale (CPS) MMSE score not provided	Living in own home Living with partners: 83.3%	Age range: 60-80	41.7
Heggestad [66], 2013	Mild, moderate an severe stage of dementia MMSE score not provided	Living in nursing homes or other care facilities	Age range: 84-94	80.0
Hulko [97], 2009	Mild, moderate an severe stage of dementia MMSE score not provided	Living in own home	Average age: 77 (74-87)	50.0
Johannessen [98], 2011	Stage of dementia: not described MMSE score not provided	Living in own home Living with spouse: 75.0%	Age range: 54–67	40.0
Keller [63], 2010	Mild to moderate stage of dementia Assessed by FAST MMSE score not provided	Living in own home Living with someone: 88.9%	Age range: 56-88	59.3
Lawrence [99], 2011	Mild, moderate an severe stage of dementia MMSE score: Range 1-29, Mean 17	Living in own home (26) Living in nursing homes or other care facilities (4) Living with someone: 73.3%	Age range: 65-96	56.7
Liou [65], 2013	Stage of dementia: not described MMSE score not provided	Living in nursing homes or other care facilities	Not described	Not described
Mazaheri [100], 2014	Moderate stage of dementia MMSE score: Range 14-19, Mean 16,5	Living in own home (10) Living in nursing homes or other care facilities (5) Living with someone (partner or child): 53.3%	Age range: 66-88	53.3
Mjorud [41], 2017	Mild, moderate an severe stage of dementia Assessed using the clinical dementia rating scale (CDR) MMSE score not provided	Living in nursing homes or other care facilities Living in special care units for persons with dementia: 50.0%	Age range: 71-95	83.0
Mok [101], 2007	Mild stage of dementia MMSE score not provided	Living in own home Living with someone: 100%	Age range: 56-80	73.3
Molyneaux [102], 2012	Stage of dementia: not described MMSE score not provided	Living in own home Living with partner: 100%	Age range: 72-83	60.0
Moyle [43], 2011	Stage of dementia: not described MMSE score not provided	Living in nursing homes or other care facilities	Age range: 70-74 to >90	68.8
Mushi [103], 2014	Stage of dementia: not described MMSE score not provided	Living in own home Living alone: 100%	Mean age: 84 (70-100)	63.4
Nowell [67], 2013	Stage of dementia: not described MMSE score not provided	Living in nursing homes or other care facilities	Mean age: 74 (62-87)	42.9
Nygård [45], 2008	MMSE score: Range 19-28, Mean 24,9	Living in own home Living alone: 100%	Age: 57-82	62.5
Öhman [104], 2005	Mild to moderate stage of dementia MMSE score: Range 15-28, Mean 21,7	Living in own home Living with spouse: 50.0%	Age range: 65-80	50.0
Pesonen [105], 2013	MMSE score: Range 14-27, Mean 20,8	Living in own home	Age: 55-68	62.5
Phinney [50], 2006	Mild to moderate stage of dementia MMSE score: Range 16-23, Mean 19,3	Living in own home	Age: 64-88	50.0
Phinney [51], 2011	Mild to moderate stage of dementia Assessed by the Global Deterioration Scale (GDS) MMSE score not provided	Living in own home	Age: 64-88	55.6
Pipon-Young [106], 2012	Stage of dementia: not described MMSE score not provided	Living in own home Living with partners: 87.5%	Age: 60-67	87.5
Rostad [107], 2013	Mild to moderate stage of dementia MMSE score not provided	Living in own home Living with partner: 75.0%	Age: 55-62	50.0
Samsi [108], 2013	Mild to moderate stage of dementia MMSE score not provided	Living in own home Living with someone: 66.7%	Age: 72-89	50.0
Svanström [46], 2015	Stage of dementia: not described MMSE score not provided	Living in own home	Age range: 80-90	83.0
Tak [39], 2015	MMSE score: Range 10-26, Mean 16,4	Living in nursing homes or other care facilities	Average age: 84.5 (72–92)	67.0
Thein [38], 2011	Moderate stage of dementia MMSE score not provided	Living in nursing homes or other care facilities	Not described	61.0
Van Zadelhoff [40], 2011	Moderate to severe stage of dementia MMSE score: Range 0-14, Mean 10	Living in nursing homes or other care facilities	Age: 68-93	Not described
Vikström [64], 2008	Mild to moderate stage of dementia MMSE score: Range 16-24, Mean 21,8	Living in own home Living with partner: 100%	Mean age: 78 (62-85)	46.2
Wolverson [52], 2010	MMSE score: Range 19-28, Mean 23,2	Living in own home Living with someone: 20.0%	Mean age: 81 (72-87)	70.0

^a^Living with someone/ spouse/partner refers to how it is described in the articles

### Data abstraction and synthesis

The principles of interpretative synthesis [60] guided the abstraction process. It focused on developing concepts based on the data from primary studies and further developing and specifying theories that integrated those concepts [61]. The analysis was inspired by qualitative content analyses [62]. This procedure enabled explicit focus on content and context in the studies, and emphasis on the similarities and differences between categories and subcategories. It also facilitated analysis concentrating on both manifest, describing what the studies reported, and latent content, referring to the interpretation of the underlying meaning.

Five phases constituted the analysing process: Pairs of authors (LKJ/EKG, KS/LHF and SE/ASH) each read and reread one-third of the papers in the *first phase*. Phrases from each paper describing lived space, in line with van Manen [12], were extracted as direct citations into a table made for analysis and sorted depending on the housing situation for persons with dementia (living in own home or in nursing home). Two of the authors (LHF and SE) then performed a further analysis. In the *second phase*, the extracted text was divided into meaning units and condensed. Condensation refers to a process of shortening and abstracting meaning units while preserving the core of the manifest content [62]. The *third phase* comprised labelling condensed meaning units with codes. Several codes were discussed in this process. This phase provided insight into the existential meaning of lived space, such as the importance of having a sense of continuity, familiarity and experiencing autonomy. Such existential elements appeared important regardless of housing situation. Comparing codes, recognising parallels and variances, and organising the codes into subcategories constituted the *fourth phase*. It became apparent through this process how the existential meaning of space may change related to the housing situation of persons with dementia. Keeping the housing situation as subcategories was therefore meaningful. Further abstraction of the codes resulted in four categories describing the manifest meaning (see Table 5: Results). In the *fifth phase*, the *comprehensive understanding phase,* the four categories with subcategories were seen as a whole, condensed and reflected upon to identify the underlying meaning of the results as one overall latent theme [62]. The third, the fourth and the fifth phase of the analysis were discussed within the author group to make consensus.

**Table 5:** Results

Categories	Sub-categories	
	Home as lived space	Long-term care as lived space
Belonging	- “the end of an era”, the loss of one’s cherished home, and the people, activities, objects and experiences associated with living at home [36] - Home as a locus of familiarity and constancy [37] - Home as a site for the expression of personal interests, values, achievements and status [37] - Home as a repository of memories of Life History [37] Wish to stay in the familiar housing situation as long as possible [91] The participants stated they had strong ties to their home [49] - The sense of “being here” (at home) was shaped over time and mad them feel strong sense of connectedness [49] A participant with dementia was clear about her wishes for the end of life: “The last thing I want to happen is to be moved. I want to feel at home.” [34] - Staying in one’s home was considered extremely important [95] To stay home was essential in order to continue experiencing a good life [107] -The person seems to be familiar with home and recognize belongings [46] - Some described their attachments to their homes mostly in the context of their interactional past and current family and social relationships [37] - Interaction with familiar people outdoors was a source of identity and social inclusion for participants [54] - The fear of unfamiliarity reduced the outside area in which the person with dementia was active [54] - Walk outside every day with our dog, even though the environment is not so familiar as before, walk in the woods find still back home [105] - Continuing life as usual with being a part of the community was seen as important for being connected to the world [105] -Outside home appears becoming unfamiliar and uncertain. Results in person with dementia being reluctant to leave home alone [46] - Participants had feelings of vulnerability and feared being exposed in public [55] -A vivid life past contribution to the society [42] -Citizenship [97] - Participants expressed that their cognitive impairments were seen as hindrances for all kinds of social activities outside their home - The importance of social location [97] -Not going out (of reason not mentioned) [102] -Cannot go out on her/his own, like go shopping, meet friends at a café [102] -Still want to go to library, and do it, even if the person with dementia has problem to act there, I might be embarrassed, but felt it stupid not to go [105] -Slipping away from the world – lived space reduces [50] -She continues to do those things she has always enjoyed- keeping the house clean, visiting with friends, taking trips, going for walks, and attending concerts. As a result, her life has changed little and she continues to feel herself as independent and responsible woman- such activities are constitutive of her personhood [51] -Outside home appears becoming unfamiliar and uncertain, results in person with dementia being reluctant to leave home alone [46] -Feelings of being left alone in the world, and to not being a participant in the world [46] - Major barriers to getting involved in activities included limited activity choices; impairment in physical functioning; and lack of accommodation in the schedule, resources and transportation [39]	- Signalled the overall “winding down” of their lives [36] The younger participant living in residential care felt out of place when receiving care in older settings [90] Relocation was symbolized as: - Familiarity with both place and activity was important to be able to perform activities independently [55] Participants expressed they were disoriented by the move [93] - Patients expressed they were disturbed by the sounds that they could hear but not clearly identify [93] - Being in an unfamiliar space with strange people caused some of them to feel anxious [93] - Being in an unfamiliar place was associated with feelings of stressfulness and confusion [54] - “Nobody seems to understand, but it’s visual stuff, visual clutter. When I was visiting last year in a dementia ward, was not only obviously the sound level, the TV and the radio and the staff talking loudly to each other, but it was a smaller area, there were lots of people, lots of tables, people coming in and out and then the occupational therapist had made stuff, which was hanging everywhere and it was just…And then there were loads of those walkers everywhere; it was just visually … really, really stressful. I would just go there for an hour and I’d be exhausted, And I often think no wonder people in nursing homes are just sitting there like that, because I felt like that when I went in, that I wanted just to sit, close my eyes, because it was too much.” [34] -Homesickness for the home [66] - This is not a home [66] - In order to protect and enforce the dignity of persons with dementia living in nursing home, they should be confirmed as whole and as individual persons, and should try to make nursing home less institutional and more home like [66] -They describe feeling of homesickness. Not being able to see the institution as a home. “A home is a place where you can walk around and do what you like. Where you don’t have to be afraid of what others think about what you are doing.” [66] - Living among strangers in the nursing home, increases the feeling of homesickness [66] - Homesickness for the home [66] - Want belonging [66] Mirror’s the way we are [63] - Same setting –lived space [63] - Helping to maintain their existing identity [102] - Disillusioned by the care environment, the noise and busyness and the lack of opportunity to engage in activities [43] - Forgetting places [103] - They have reflective thinking about the ward environment, and rules/ restrictions [67] - Familiarly surroundings supported and encouraged occupations, was the key to activity [104] - She will still be able to sense, feel and appreciate the place where she is [51] Maintain a sense of self, remain the same by doing and going the places that he has been used to [106] You get to start a new life (in the care home) [38] -I miss my old home. We are miles apart (the husband and wife living in the care unit, but different rooms) [38] -I want to go back to my own home, to be with people I know [38] -Settle in the new homes (long term care homes) seemed to be a result of pre-visit before moving, continued contact with family, being accepted by others, company, care and assistance from staff [38] -I can go to my room when I like, …..I prefer to stay in the living room with the others [40] -I prepare to have my private living and my privacy. I would like to have my own house [40] -I think it is this country, I wouldn’t like to live anywhere else, (So I suppose I find hope in my country and family) [52] -As long as you are not home, it cannot be better than this [41] -Living in the nursing home as a temporary solution [41] - It is…you know how it is, in a nursing home [41] - Make it homely to feel less lonely [41] - They [the other residents] just sleep… just sit there and cry and sleep and cry… [41]
Meaningfulness	- Home as a place of retreat, solitude and rejuvenation [37] - Home as a site for the expression of functional competence and engagement in meaningful activities of daily living [37] - Home as a centre of socialization, connectedness and affiliation [37] - Participation in day care program implied a chance to experience a more meaningful social life [90] - Getting upset by misplacing things. Wanting the house to be neat, clean and well-organized [100] - Being ashamed of lost competence and blamed themselves for the practical consequences of their conditions like misplacing household items and not being able to locate them when others needed them [100] -Notes and reminding messages from spouse and family keeps me busy, I have something to do – in the community, outside home, in church [50] - I like retirement. I like being at home, ….. mostly we do things together (mutual decision making) (phase 1) [108] -Over time the person with dementia (and carer) described their lives as having shrunk – they were doing less (restrictive decision-making (phase 3)) [108] -Living space important for the way in which their apartment enable to attend to socio-emotional preoccupations (visitors, discussions, possessions, view, independence) [97] -Expected them self to be able to use the technology they had at home [45] -Home gave them also frustration and anger at the experience of being idled [107] -They sat and waited for something (at home?), but they did not know what they were waiting for [107] -I like retirement. I like being at home, ….. mostly we do things together (mutual decision making) (phase 1) [108] -Daily life as described in the conversations appeared to be uneventful where the person with dementia did not seem to take the initiative to do anything at home other than sit and look out of the window, browse through newspapers and magazines, watch TV or go to bed [46] -I sit here (at home) like a crow in her nest [64] - Participants reported their use of public space was constantly changing. Meaning the public space that participants found comfortable had gradually become smaller [55] - Participants expressed the value of being able to perform activities and visit different places as it created a sense of being an active and independent person who is part of society [55] - The main reason to perform activities in public spaces was that they found it important to be able to do as much as they could, because they knew that AD is progressive and that their ability to perform activities in the future would change [55] - Living close to services such as stores or churches was expressed as important [49] - Patients expressed that being able to see the “outside world” was important [94] - Going out was seen by some as an opportunity for enjoyable informal encounters with friends and neighbors [54] - Getting out of the house was important to prevent loneliness [47] -Disappointed of not being supported in doing activities like outdoor walking [42] -Instrumental preoccupation among the more marginalized participants can be seen in daily-life activities such as cooking and walking [97] -Progress in the disease became hindrances in performing of activities giving life space, had to stop driving and walking alone -The change seemed to be accepted [104] -The person is managing dementia by keeping active in the world. But may have a need to overcome feelings of shame sometimes [105] -Coping strategy: Handling orientation problems by walking to the bakery nearby home in the morning to buy the smallest item in order to get a receipt with the date on [45] -People with mild to moderate dementia did not disappear or retreat from activities outside home or at home, with help from their families they found ways to stay involved both in everyday activities inside the home and out in the community [50] -No I do not go out on my own any longer. My wife does all the shopping [64] -To get out of the house, … getting about, … = keeping busy experiences important [52] - Their interest had changed from the ones they had in the past, this influenced the activities they engaged in in the public space [55] - The main reason to perform activities in public spaces was that they found it important to be able to do as much as they could, because they knew that AD is progressive and that their ability to perform activities in the future would change [55] - Being able to go out and do ordinary daily activities was expressed as being extremely important [49] - Being able to go out was significant in the lives of the participants [54] - Functional reasons for enjoying a walk outdoors included exercise and the benefits of breathing fresh air [54] - Walking outside ensured emotional well-being. Some also mentioned a sense of relief, escape and freedom [54] -Taking walks in the familiar environment helped them maintain sense of coherence to stay active and maintain the current status [104] -Walk outside every day with our dog, even though the environment is not so familiar as before, walk in the woods find still back home [105] -He had stopped running marathon, play the piano in church, doing work around the house [50] -As long as I can get about, and I’ve got my health (I have hope in life) [52]	- Participants indicated that coming to day care resulted in a state of contentment and increased energy [90] - One resident: Being thankful if he could rest in bed all day [26] -White British more positive about caring homes, frequently identifying value in enabling PWD to avoid depending on loved ones in the later stages of the illness [99] - Participants primarily depended on activities provided by the nursing homes [39] - Walking inside and outside of the facility on a daily basis was an important individual activity to residents [39] - Residents played bingo to meet other people and expand social opportunities [39] -I want to go back to my own home, to be with people I know [38] -I always do the washing here, I always did this at home as well….. (giving a feeling of home?) [40] -I can go to my room when I like, …..I prefer to stay in the living room with the others [40] - Participants expressed a lack of activity and that they felt bored in the residential care home [92] - Patients expressed that the care they received was more important than the surroundings [94] - Residents with dementia found that most of the activities offered by nursing homes did not interest them. Activities were limited or did not align with their hobbies or interests [39] -Participants primarily depended on activities provided by the nursing homes [39] -Walking inside and outside of the facility on a daily basis was an important individual activity to residents [39] - If you can’t be home, you must be happy you are on your feet and can have your own room [41] - It is quiet like the grave here [41] - I have no interest in anything here [41]
Safety and security	Carry on as normal [91] By not “making mistakes” the participants were able to continue to remain at home [49] - Threats of security contrasted with a feeling of peace and comfort [49] - Participants locked their doors to secure them from the outside world [49] - The temporal meaning of “as long as I can” was limited in duration and the women acknowledged that their need to move away from their home would come at a certain time [48] - Factors that they anticipated would contribute to the endpoints of living alone were: Being trouble for family, being worse or being exhausted [48] Participants expressed their need to retreat to a place of safety, usually their home. Staying close to home made them feel safer [47] Being in the rhythm of life [97] -Worrying about others reporting that they have difficulties living on their own could force them to move out of their apartment [97] -Marginalized people did not see dementia as particular problematic. They are occupied with psychological and safety needs [97] Being able to work at home in their own pace to avoid stress and facilitate coping [98] -Preparing and using systems to facilitate everyday coping [98] Getting upset by misplacing things. Wanting the house to be neat, clean and well-organized [100] -Felling confident and secure but also isolated [100] The home created a dilemma, it was the setting of frustration but also for haven [102] - Tried to keep up with social traditions / activities like eating lunch out at Sundays [102] -Life became more home-centred [105] Everyday articles lose meaning. Their home and everyday articles does not serve as a remainder for attention to spur action anymore [46] -Home seems to constitute security [46] -I cannot manage at home any more…, it can get quite difficult sometimes [38] Experiencing getting lost, but being safely returned home. Still feeling safe and still doing activities like outdoor wandering [100] -Feeling fine and secure in going around and doing activities in the surrounding community even though they had experienced events where their ability to manage had been compromised [100] Being disorientated, misunderstanding, and forgetting. But not afraid of doing activities like visiting the graveyard, taking the train or taking a walk alone in the community [100] -Activities performed independently out of the house was not feasible [102] Would rather cuddle up safe at home, than going outside, going outside is becoming difficult [105] Maintain a sense of self, remain the same by doing and going the places that he has been used to [106] - Participants experience of public spaces was also influenced by what kind of preparation activities that were necessary [55] - Participants regarded public space to be less accessible at certain times of the day, planning what time to perform an activity was therefore important [55]	-Residential care was associated by most participants with a place of hospitality and rest where one is served meals and let “someone else do it” [36] - Expressed carers focus upon risk and danger conflicted with their need to be independent [90] - Feelings of safety and comfort were associated with familiar areas [54] It seemed important to participants with dementia that they did not wanted to be in a noisy or overcrowded environment, the important things were calm, peace and quiet [34] There was an expressed ambivalence about living in the care home. - on one hand the care home was the preferred place of care. However, the relationship to the members of staff was important for their total experiences. If the relationship were negative, then the negative impact of living in a care home was more keenly felt [96] Safe and nostalgic comfort [65] -Cleanness and feeling secure. The old style setting provided a sense of security as they grew up in a place like this. The old style made it easy to new clients to acclimate to the environment [65] Enjoy to not have responsibility of housework and food preparations in these environments [43] It is expected that the care homes provide them with company, make them safe, relief them for day-to-day housework and care for them [38] -that you are not strong enough to live at a farm…and do everything that needs to be done [41] - Yes, I would rather be at home, but I probably couldn’t. I am so old (crying) that I couldn’t do anything anymore [41]
Autonomy	- Home as a locus of autonomy, Control, choice and freedom of action [37] -Valuing travelling, car and having fun. The loss of previous valued activities as ability to drive [42] - The importance of being able to take care of oneself [97] -Expected them self to be able to use the technology they had at home [45] - Increasingly difficulties noticing and using signs and maps limited their access to places in public space that were not familiar [55] - Participants felt that replacement of service personnel with everyday technology influenced their accessibility [55] - Participants access to medical care was limited because it required communication with answering machine on the telephone [55] - Difficulties in finding one’s way in the public space were seen as a serious obstacle by the informants, subtle changes in landmarks in the public space influenced their perceived accessibility [55] -Staff taking a person out of the ward to smoke, express gratitude [67] -Walking outside ensured emotional well-being. Some also mentioned a sense of relief, escape and freedom [54] Others concerns about them getting lost while driving or walking [97] -Loss of employment and the ability to get different places [101] -Cannot go out on her/his own, like go shopping, meet friends at a café [102] -Being able to go out on one’s own when desired gave a feeing o freedom, experienced pleasure, partly giving autonomy [104] -Bike cycling in the town had stopped due to traffic, but short tours on the bike around the summerhouse was appreciated and gave freedom [104] -Driving the car, gave a feeling of freedom and wellbeing [104] -Loss of driving license increased dependency, cannot freely change space [107]	- Meant a shift from living an active and independent life in one’s private residence to cohabitation with other older people in a more structured, protected, supportive and collective living environment [36] - The participants expressed living with dementia in residential care was difficult, a situation over which they had little control, had feelings of loss and uncertainty and felt they were isolated from their family and expressed they were lonely [92] - Participants did not look forward to moving away and being there (in the nursing home) [49] - Going to a residential home was seen as “giving-up” and associated with the loss of freedom the participants had whilst living independently [47] - Participants feared going to residential care [44] - Participants highlighted they wanted to be involved with shaping their service, work together with their provider, share information and having their needs appreciated [44] -Feeling restricted by the environment at a care home. Bing monotonous, with little to do and poor quality interactions [26] -The residents feel that their freedom is restricted: It like being in a prison without bars (the doors are locked e.g. The door to the kitchen, the worker’s office etc.). Cannot just go and get a glass of milk in the kitchen [66] -Like being in a prison [66] -Want freedom [66] -The experience of lack of privacy e.g. Own seat [65] -The experience of hospital-like environment created anxiety and discomfort like: I am not sick; why do I have to stay here [65] -Previous enjoyment with partaking in outside activities, such as walking. Now: Activities were restricted, controlled by staff, not allowed outside the care facilities => kept away from new life experiences, natural beauty and growth [43] -The ward system –that environment interact with the personhood [67] -They have to respond (live) within the restrictions of the ward, a kind of trapped in the situation [67] -Lack of individual choice [67] -If I just could be trusted to go out and smoke [67] -Staff taking a person out of the ward to smoke, express gratitude [67] -They have reflective thinking about the ward environment, and rules/ restrictions [67] -Person with dementia, she does not want to be put away or placed at a nursing home if she cannot take care of herself [50] -I could go out whenever I wanted at home (previous home), but now don’t do much, have to wait for my son to take me out [38] -I would like to not go (to the long term care home) …. You cannot do what you want…. You have somebody over you…… [38] -Restrictions in relation to outdoor walking. Dependent on others to go out [66] -Lack of control over their environment [43] - if you come to a place, you must put some [effort] into it, [and put some things] behind you. I live here, I will be content here [41]

## Results

The material revealed four main categories describing the experience of space: (1) *belonging*; (2) *meaningfulness*; (3) *safety and security*; and (4) *autonomy*. The analysis showed that *living in one’s own home* and *living in long-term care* involved different settings and posed diverse challenges for the experience of lived space among persons with dementia. Therefore, the two settings constitute two subcategories. The descriptions were distinct and associated with the setting. The studies described the space of home or long-term care itself but also the space surrounding the place they lived (i.e., outdoor space). Some studies also included descriptions of the experience of public space and national space.

### Belonging

#### Own home

Persons with dementia considered living at home and in their own home to be very important. They perceived home as a place for belonging and the key for living a good and meaningful life. They described their experience of home as feeling at home and being at home. Feeling at home included the feeling of belonging and being a part of something. Being at home represented the experience of being together with important people in their lives. It also embodied specific familiar objects, activities and experiences concretizing values, interests and status. Home and the symbol of home connected persons with dementia to their history. One participant said, “It mirrors the way we are” [63]. Engaging in social activities, being with friends and family, and attending socio-emotional preoccupations were important aspects of their experience of home. They described doing things with others in positive terms.

However, some persons with dementia experienced their daily life as uneventful, as they were not able to take the initiative to do things. They described that they sat around and did nothing; some described the experience of being idle. One participant said, “I sit here (at home) like a crow in her nest” [64].

In a wider sense, belonging at home also incorporated different levels of belonging to outside environments. The experience of being able to use the local neighbourhood and take part in the community and the feeling of belonging to the country were considered important. This contributed to a feeling of connectedness with the world. Outdoor environments were considered arenas for social activities and an essential source for sustaining identity. Familiarity with outdoor environments also seemed important for the ability to carry on with life and to maintain known activities. Being a part of a neighbourhood could prevent loneliness, encourage social activities, make living a typical life possible and lead to a more vivid life. Living close to sites such as churches or stores was therefore important. Nevertheless, some also described the experience of being vulnerable and the fear of being exposed when in public spaces, and others used terms such as being embarrassed or feeling shame.

The feeling of unfamiliarity increased with the symptoms of dementia. In cases of concrete episodes of disorientation, persons with dementia perceived that outdoor space was automatically narrowed. Their ability to use public places decreased due to the development of dementia. For instance, not being able to drive increased this experience. In addition, they experienced outdoor environments as unfamiliar and their possibilities of participation as restricted. They described this experience as slipping away from the world and being left alone. Some stated that they managed to find their way back home even though the environment was less familiar than before; others were reluctant to leave home alone.

#### Long-term care

Persons with dementia living in long-term care described belonging as the experience of being familiar with the setting and being in the right place. Relocating into long-term care appeared to cause disorientation in some cases and challenged their overall sense of belonging. They described relocation in two ways: either as the beginning of the end or as the start of a new life that signalled an overall “winding down” [36].

Belonging, as an experience of being part of the new environment, appeared to be important. In some studies, persons with dementia reflected upon the long-term care environment and what would promote a sense of belonging. Persons with dementia were still able to sense, feel and appreciate lived spaces. However, the process of being familiar with a new place after relocation and experiencing belonging appeared to be time consuming and dependent on several influences. Being able to maintain contact with family and to uphold familiar activities were emphasized as important. Becoming familiar with the long-term care setting and its associated activities and being accepted and confirmed as a whole person by the other residents and health care personnel were essential aspects of developing a sense of belonging. Being in an unfamiliar setting with unknown people appeared to foster stress, confusion and anxiety. Lastly, some persons with dementia emphasized the ability to have a private life as important to sustain their experience of belonging to the place.

Persons with dementia who struggled to find their place and to experience belonging in the new environment related these difficulties to a number of issues. Some described their inability to view the long-term care facility as their home. Some also expressed being disillusioned by the noise and busyness in the long-term care environment, and they described the care environment as a place with rules and restrictions, which restrained their individuality. They experienced the long-term care setting as uncomfortable. Several studies described their struggle of homesickness, which appeared to be related to both the difficulties of experiencing hominess and belonging in the long-term care environment and to the longing for familiar others, their own home and familiar surroundings. Living everyday life among unfamiliar residents seemed to increase their feeling of homesickness.

### Meaningfulness

#### Own home

Persons with dementia described home as a centre for meaning. They described it as a place for retreat, solitude, and rejuvenation; a centre for socialization, connectedness and affiliation; and a centre for meaningful activities of daily living. Home was supposed to be organized and presented in a particular manner. Some described the importance of a clean and neat home, even if it was sometimes described as difficult to keep up with the preferred standard for their home due to their dementia symptoms. Some persons with dementia felt ashamed when they were not able to take care of their home the way they wanted to and had before, for example, when next of kin took over all of the shopping. Persons with dementia described that life had shrunken after dementia came into their lives and that they felt that they were dependent on their next of kin. However, one woman described that her life had changed little after she received her dementia diagnosis because she had continued to do the things she used to, and she still felt like an independent and responsible woman.

Doing outdoor activities was considered central for the experience of meaning, even though some experienced that their interest in certain activities had changed after developing dementia. Some emphasized that it was important to do as much as they could do for as long as they were able to. Going outside, for instance, for a walk in a familiar environment allowed them to maintain a sense of coping and current status. To some, this was even an expression of hope in life. Going for a walk outside their home not only ensured physical exercise and fresh air but also created a sense of well-being. Additionally, when the environment was less familiar than before, going outdoors for a walk alone was important to participants in some of the studies.

#### Long-term care

Meaningfulness was related to the person’s ability to be occupied with interesting and relevant activities when living in long-term care. Some persons with dementia experienced long-term care as an important arena for social activities and a place where they could meet people and expand their social opportunities. However, to others, relocating to long-term care had the opposite effect, and they longed for privacy. Some also expressed that they were bored in this setting, that the long-term care lacked alternatives for activities, and that they longed for their own home and well-known activities.

Although their experiences clearly varied, many persons with dementia experienced that they were dependent on health care personnel to initiate activities. However, there was variation in how they experienced meaningfulness in relation to participating in the activities. While some expressed that they wanted to be left alone or preferred to stay in bed all day, others described that socializing with others led to a state of contentment and increased energy. Examples described in the articles were individual activities such as walking inside and outside the facility or doing familiar things such as cleaning and socializing (through, for instance, playing bingo or staying in the living room). Others stated that the activities offered in long-term care were limited or did not interest them. Others emphasized that they could choose whether they wanted to participate in activities. Persons with dementia highlighted that being familiar with the place was the key to empowering their maintenance of daily activities and retaining some sense of independent living.

### Safety and security

#### Own home

Persons with dementia described home as a place for safety and security, a place where they could avoid stress and do things at their own pace. They also described home as an arena for coping, comfort and continuity in relation to traditions and social life. It was important for them to carry on as normal. However, home was also a setting for frustration and anger. With dementia, life became more home-centred and isolated. Some expressed a bad feeling of no longer being able to manage living at home because, for instance, everyday articles lost their meaning. It was also described as important to prepare and use systems to facilitate everyday coping as long as possible.

Some persons with dementia stated that continuing to maintain outdoor activities and to use public places was a useful coping strategy for handling their cognitive impairment. Others described that their feeling of anxiety constrained their outdoor activities. Some locked the door to the outside world in order to protect themselves and feel safe and secure.

Several studies emphasized the thoughts of persons with dementia about no longer being able to live in their own homes/in familiar places. “To stay home as long as I can” [48] was acknowledged as limited in duration. Phrases such as “to stay at home” [47] and “being moved from home” [34] were used to describe the possibility of going to long-term care. Some expressed worries about others reporting that they had difficulties living in their homes alone. They tried to avoid “making mistakes” [49] and attempted to hide their difficulties from their next of kin. Some expressed that the burden on their families, their worsening condition and their family members’ exhaustion were factors facilitating the endpoint of their living in their own home.

In some studies, persons with dementia emphasized the importance of being outdoors despite getting lost, misunderstanding and forgetting. Some still felt safe and were not afraid of doing activities. They trusted that someone would find them, take care of them and lead them home. Doing activities on their own and going to well-known places allowed them to maintain a sense of self. In other cases, performing activities independently and out of the house was infeasible. Others preferred staying in their homes to feel safe.

#### Long-term care

Safety in long-term care was related to the sense of being safe and comforted. Persons with dementia described long-term care as a place for hospitality and rest, as it relieved them of household responsibilities, cleaning and cooking. It was also expected that they were cared for and provided with company when residing in long-term care. Familiar, calm and peaceful surroundings were associated with a feeling of safety and comfort. They described an “old-style” [65] interior setting as a way of providing a sense of security and continuity.

Living in long-term care facilities were described as providing an experience of safety and security. Safety and security were emphasized in numerous studies. In some cases, persons with dementia expressed a need to feel secure and safe while living in long-term care. However, in other studies, they highlighted the tension between their need for safety and security and their need for independence.

### Autonomy

#### Own home

Many persons with dementia perceived that living in their own home was the locus of autonomy, control, choice and the freedom to act. They experienced home as a place for preserving autonomy, and they highlighted the importance of being able to take care of themselves and to use aids such as technology. They expected themselves to be able to use helpful technology. The loss of former and valued activities, such as the ability to drive, was described as difficult.

Being outdoors alone and being able to drive provided autonomy and were experienced as a sense of relief, escape, wellbeing, pleasure and freedom. In the studies, persons with dementia described restrictions related to going out on their own, being dependent on others to go out, a loss of ability to go to new places, and others’ concern about their getting lost. Some persons with dementia also emphasized the importance of accessibility to the outside environment and said that signs and landmarks were promoting factors. It was often difficult for them to find their way in a public space, and using a map did not help. In addition, public spaces may be less accessible at certain times of the day. Another obstacle to accessibility cited by persons with dementia was the replacement of service personnel with technology. They could no longer just buy a ticket from service personnel, as tickets were to be bought from ticket machines. The loss of the possibility to go out and to drive increased their dependency and prevented them from moving around freely.

In studies focusing on the relocation from home to long-term care, several persons with dementia described the experience of losing their autonomy. Relocation was not their preference, and they did not look forward to it; it was paralleled with “giving up” [47], “being putted away” [50], and “letting somebody be over you” [38]. They also experienced relocating to residential care as a shift from being an active and independent person to living in a more structured, protected and supported life collectively with others.

#### Long-term care

Lack of autonomy was related not only to relocation but also to everyday life in long-term care. Living in long-term care was associated with monotonous living, a loss of abilities and freedom, fewer opportunities for individual choices, a lack of privacy and uncertainty. Some experienced that they had poor social interactions and felt isolated from friends and family.

In several studies, persons with dementia emphasized that in long-term care, they were involved in shaping their own services, were informed and had their needs appreciated. However, in some cases, persons with dementia experienced having little control over their own life. They experienced restrictions in relation to the long-term care environment, the organization of the ward and the rules and routines operated by the staff. They described that being dependent on the staff to be able to maintain well-known activities and being held indoors (not being allowed to go outdoors) inhibited their autonomy. In one study, living in long-term care was described as “living in a prison without bars” [66]; another study described a feeling of being trapped [67].

### A space where the walls keep closing in

The comprehensive understanding of the categories described is captured in the latent theme: “Living with dementia is similar to living in a space where the walls keep closing in.” Our findings show that lived space is reduced as dementia develops. This indicates a process whereby lived space gradually becomes smaller. A space where the walls keep closing in can be understood through the metaphor of the Russian “babushka doll,” which is a set of dolls of decreasing sizes that all fit inside one another one by one. Similar to the “person within a similar person” of the babushka doll, people with dementia experience the walls closing in, and step-by-step, the experience of lived space is reduced from large and wide to small and restricted. Thus, the findings indicate the strong connection between the experience of lived space and the importance of sustaining feelings of belonging, meaningfulness, safety and security and autonomy among persons with dementia. All of these aspects can be considered existential in nature. These are all feelings that might support the ability to preserve a sense of continuity, maintain self-identity and sustain a sense of attachment to a place.

## Discussion

The aim of this systematic meta-synthesis was to interpret and synthesize the experiences of space in persons with dementia. The main findings indicate a process whereby lived space continually decreases due to the progression of dementia. The metaphor of the Russian babushka doll can be used to describe the experiences of persons with dementia living in a space where the walls keep closing in. Regardless of the progression of dementia, they continue to experience the spatial dimensions of life through lived space. According to van Manen [12], lived space is one of the cornerstones of the lifeworld experience, and it influences and is influenced by other lifeworld perspectives.

Research within the field of environmental gerontology, i.e., [13, 68–70], has emphasized the importance of supporting the ability to preserve a sense of continuity, maintain self-identity and sustain attachment to a place for maintaining a sense of being in place, or, in van Manens’ [12] words, maintaining a sense of space. Our findings show that belonging, meaningfulness, security and autonomy are essential elements of the experience of lived space among persons with dementia. Despite the differing use of the terms, the essence can be understood as compatible with the findings in our study.

With the progression of dementia, patients’ cognitive abilities decrease [71]. As indicated in this study and others, i.e., [18, 29–31, 72], the diminishing cognitive capacity might challenge persons’ capability of familiarizing themselves even with well-known environments, making their own choices and taking advantage of possible opportunities. Due to progression of dementia, persons’ physical capability will also often change. This *change of lived body*, in accordance with van Manen [12], leads to a decrease in the room of action; both the experience of the space and the environment grow smaller. However, to maintain safety and security, meaning, belonging and autonomy as essential dimensions of space, it appears that a reduction of the environment is a necessity.

The progression of dementia often leads to a need for relocation from one’s own home to a long-term care facility [73]. Thirteen studies described participants’ experiences of lived space while living nursing home or other facilities. Within our data, moving into a long-term care facility was described as “giving up”, a loss of freedom, a lack of privacy, being in prison and living a structured, protected life in a collective living environment. Fear of relocation was often expressed long before it actually occurred. This fear can be understood as a fear of facing a new and unknown life situation and as a fear of being taken away from a well-known daily life. The replacement of the lived space might threaten all of the essential dimensions of belonging, meaningfulness, safety and security, and autonomy. One might see lived space as an expression of existence. According to Goyal et al. [74], anxiety symptoms among persons with dementia could be a reaction to loss and worry, especially the experience of dealing with a new situation, i.e., relocation. Goyal [74] stated that anxiety among these persons must therefore be understood as existential in nature. Anxiety symptoms are common among persons with dementia and might lead to negative impacts such as decreased function in activities of daily living [75], increased dependency [76] behaviour problems [77, 78] and an additional burden on the patients and caregivers [79]. Nonetheless, anxiety symptoms in patients with dementia are often overlooked by caregivers and health care personnel [80].

The results of this study suggest that facing new environments threatens a person’s existence, such as the ability to uphold a sense of control over one’s own life, protecting privacy, and making choices of importance. When relocating into a residential facility, persons need to reconstruct their sense of space. They have to convert a place that is neutral into a place that has meaning in the context of their ongoing life [13–15]. Lived space is closely related to a person’s maintenance of his or her self-identity and sense of attachment to the place [13–15]. The metaphor of the Babushka doll pictures the recognition of oneself, being oneself and feeling connected to one’s self-identity despite decreasing environmental space.

Several studies described the outdoor environment as an important factor for lived space while living in one’s own home. Going outdoors was connected to emotional well-being, prevention of loneliness, being physically active and having a feeling of freedom. To some, doing the same things and going to the same places as before helped them maintain a sense of self. Taking part in the local environment and community was also described as central to some. Others described that going outdoors was difficult due to the orientation and memory impairment and they felt isolated in their own homes. In most of the studies that interviewed persons with dementia living in their own homes, the outdoor environment was a topic even when they did not make use of it. In contrast, this was not emphasized in most of the studies interviewing persons with dementia living in long-term care facilities, where outdoor space was not a topic at all.

In our study, persons appeared to have adapted themselves to their situation as a conscious or unconscious strategy by reducing their environment. As dementia progresses, persons become increasingly dependent on others. This includes help with outdoor activities, finding one’s way around and taking the initiative to go outdoors. Whear et al. [81] stated that persons with dementia living in long-term care facilities often spend all of their time or most of their time in doors. Others have found that spending time in a garden could have an impact on the agitation in care home residents [82]. Even so, being outdoors in nature or a garden might have positive consequences for persons with dementia. In a review, Gonzalez and Kirkevold [83] found that targeted use of plants might have a positive influence on the function, behaviour and well-being of people with dementia.

The experience of lived space can be seen in relation to the concept of ‘at-homeness’, which refers to a place where the person feels safe, connected, respected, understood and loved [84]. Öhlen et al. [85] shows in a review how older persons construct ‘at-homeness’ despite illness and disease as a particular aspect of wellness. At-homeness is a feeling that is created in partnership with others. The maintenance of meaningful social relations, meaningful *lived relations*, are therefore considered important dimensions of space and as prerequisites for feeling at home and being connected to place, whether the persons are living in their own homes or in long-term care facilities [33, 86, 87]. The frameworks of person-centred dementia care emphasize the importance of sustaining social needs [88]. Based upon these values, McChance et al. [89] describe the importance of setting individuals free in a flourishing environment where they are confirmed and respected as unique individual persons, whether they are living at home or in a nursing home.

### Strengths and limitations

We performed a systematic meta-synthesis with transparent descriptions of the selection process for the included articles. However, we acknowledge that a complete overview was not attainable. The value of both individual reviewers and the use of pairs of researchers to evaluate the studies should be acknowledged.

The systematic approach taken to source and analyse the available qualitative data is a considerable strength of this meta-synthesis. Qualitative content analysis facilitated explicit attention on the manifest descriptions of the experiences of persons with dementia provided through the primary studies. Through combining manifest descriptions with interpretation of the latent meaning of their experiences, we believe that this meta-synthesis provides new and important contribution to the field. However, data comprising descriptions of people’s experiences always involves multiple meanings depending on subjective interpretation. The dialogue among the authors throughout the analysing process was therefore valuable to seek agreement of the way in which data was sorted and labelled. Together with a high level of transparency through the rich presentation of findings and the condensed meaning units provided in Table 5, this contributed to the credibility of the study. To facilitate transferability, providing description of the context of study was important. Classification of dementia severity among participants was important to appraise dementia progression in relation to experiences of lived space. However, it appeared difficult because several articles lacked descriptions of dementia severity. The synthesis of results depending on the participants’ housing situation still provided valuable insight into how progression of dementia may influence the experiences of lived space. Adopting the same search strategy as the earlier systematic review, which studied the experience of the lived relation in persons with dementia [5], has enabled direct comparison for a more in-depth understanding.

Only studies published in scientific journals were included in the systematic review. The voices of persons with dementia published in the “grey literature” were not explored in this article. An analysis of the different interviewing guides could explore different views of persons with dementia and were not always fully described in the included studies.

## Conclusion

This meta-synthesis revealed four main categories: (1) *belonging*; (2) *meaningfulness*; (3) *safety and security*; and (4) *autonomy*. The categories illustrate how the experience of lived space may change with the living situation of persons with dementia. Through interpreting relocation to nursing home as a measure of dementia progression, our findings show that persons with dementia experience a reduction in their lived space as dementia develops. This indicates a process where lived space gradually becomes smaller. The Russian babushka doll serves as a metaphor describing the comprehensive understanding of the categories leading to the latent theme: “Living with dementia is like living in a space where the walls keep closing in.”

This meta-synthesis indicates the importance of being aware of the experiences of the spatial dimensions in the lifeworld of persons with dementia. To sustain person-centred care and to support the preservation of continuity and identity, one has to acknowledge not only the physical and social environment but also space as an existential experience for persons with dementia.

## Abbreviation

CASP: Critical Appraisal Skills Programme
